# Ventilatory Efficiency in Children and Adolescents: A Systematic Review

**DOI:** 10.1155/2015/546891

**Published:** 2015-05-03

**Authors:** Paloma Lopes Francisco Parazzi, Fernando Augusto de Lima Marson, Maria Angela Gonçalves de Oliveira Ribeiro, Camila Isabel Santos Schivinski, Jose Dirceu Ribeiro

**Affiliations:** ^1^Department of Pediatrics, Faculty of Medical Sciences, State University of Campinas, Tessália Vieira de Camargo, 126 Cidade Universitária “Zeferino Vaz”, 13083-887 Campinas, SP, Brazil; ^2^Department of Medical Genetics, Faculty of Medical Sciences, State University of Campinas, Tessália Vieira de Camargo, 126 Cidade Universitária “Zeferino Vaz”, 13083-887 Campinas, SP, Brazil; ^3^State University of Santa Catarina, Center of Physical Education and Sports, Coqueiros, 88080-350 Florianópolis, SC, Brazil; ^4^Department of Clinical Medics, Faculty of Medical Sciences, State University of Campinas, Tessália Vieira de Camargo, 126 Cidade Universitária “Zeferino Vaz”, 13083-887 Campinas, SP, Brazil

## Abstract

*Introduction.* The index of ventilatory efficiency (VE/VCO_2_) obtained by the progressive exercise test has been considered the gold standard in the prognosis of adults with heart failure, but few studies have evaluated this approach in children. *Objective.* To verify the scientific evidence about the VE/VCO_2_ in pediatric and adolescents patients. *Methods.* A systematic literature review was carried out using the key words VE/VCO_2_, children, and adolescents using the PEDro and PubMed/MedLine databases. Clinical trials published from 1987 to 2014, including children, adolescents, and young adults up to 25 years, addressing the VE/VCO_2_ index as a method of evaluation, monitoring, and prognosis were considered. *Results.* Initially, 95 articles were found; 12 were excluded as the title/abstract did not contain the VE/VCO_2_ index or because they included patients greater than 25 years of age. From the remaining 83, 58 were repeated between the databases. The final sample consisted of 32 studies including healthy children and children with respiratory and other diseases. *Conclusion.* There are few studies involving cardiorespiratory assessment by ventilatory efficiency. The studies highlight the fact that high VE/VCO_2_ values are associated with a worse prognosis of patients due to the relationship with the decrease in pulmonary perfusion and cardiac output.

## 1. Introduction

Maximal and submaximal cardiopulmonary exercise testing on a treadmill or a cycle ergometer is frequently used in adults and children to evaluate the performance of the cardiac, vascular, respiratory, and metabolic systems in healthy individuals and patients, including those with chronic illnesses [1, 2].

The tests relate to several exercise tolerance mechanisms evaluated by different cardiopulmonary parameters. These parameters include respiratory frequency (FR), heart rate (HR), maximal oxygen consumption (VO_2_peak) and, more recently, the relationship between ventilation and carbon dioxide exhalation (VE/VCO_2_), which represents the analyses of the relationship between minute ventilation (MV) and carbon dioxide production (VCO_2_) or the ventilatory equivalent for CO_2_, that is, the amount of air necessary during expiration to eliminate one liter of CO_2_ [2, 3]. Some of the parameters do not have normative values for the pediatric population [2].

The VE/VCO_2_ index defines ventilatory efficiency, for it reflects the interaction between pulmonary ventilation, pulmonary perfusion, and cardiac output, contributing to the prognosis of the patient.

An elevated VE/VCO_2_ index, which is present in patients with cardiac diseases, indicates inefficient ventilation [4]. Serra (2012) states that the clinical and functional condition of the adult patient with chronic cardiac insufficiency, as well as their prognosis, is progressively aggravated as the MV rises to match the production of VCO_2_. In patients with chronic cardiac insufficiency, high VE/VCO_2_ index numbers are associated with increased mortality; in other words, amounts that are equal to or greater than 45 imply a 50% mortality rate in two years, which can indicate a need for a heart transplant [5].

To some authors, the VE/VCO_2_ is the only mortality predictor in adults with coronary diseases, being that the rise of the VE/VCO_2_ index is attributed to poor pulmonary blood diffusion and increased ventilation in the physiological dead space [6, 7]. Index numbers just as high may occur in severe pulmonary diseases [5].

In the pediatric population, the VE/VCO_2_ has been studied in different clinical situations [8–22]. However, it is clear that the application of the VE/VCO_2_ index in this particular population needs more research. Meanwhile, the published studies must be meticulously analyzed for a systematization of our knowledge up to this point. Thus, the objective of the present study was to verify the scientific evidence on the VE/VCO_2_ index applied to children, adolescents, and young adults and to discuss the results.

## 2. Methods

Research was done using the PEDro, Physiotherapy Evidence Database (http://www.pedro.fhs.usyd.edu.au/accessed on February 18, 2015), and MEDILNE, Medical Literature Analysis and Retrieval System Online/PubMed (accessed on February 18, 2015) databases. The selected articles ranged from 1987–2015, corresponded to the preestablished inclusion criteria, and were published in the following languages: English, Portuguese, and Spanish. Two evaluators made the initial study compilation and consensually elected the compatible studies for the abstract analysis. The second step in the analysis consisted of evaluating the compatibility of the abstract with the required criteria; the manuscripts were selected for complete reading and possible inclusion in the study.

The authors elaborated on the strategic search through the databases, as presented in Table 1. The inclusion criteria for this review covered all the studies involving patients 25 years old or younger, regardless of diagnosis, who used the VE/VCO_2_ index as an evaluation, monitorization, or prognosis tool.

As presented in Table 2, in the PEDro database, which is specific to physiotherapy, there were a limited number of studies published in the last few years and, according to the scale of points, which evaluates methodological quality, the studies performed poorly. The studies from the PEDro database received 3.0–5.4 out of 10 points; therefore, they were considered to be of low-quality from a methodological point of view.

## 3. Results

**Figure 1:**
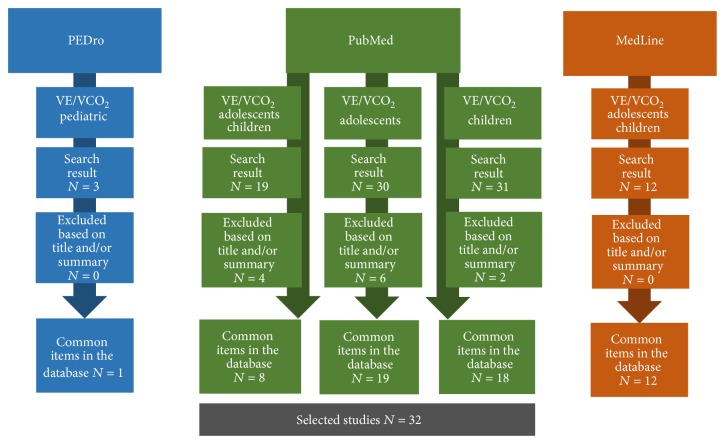
Flowchart of the study selection.

The initial search presented us with 95 studies. Eighty-three were selected (using the title) for a more specific analysis of the abstract, although 49 were repeated between the databases. After this analysis, 32 studies were selected for full reading and inclusion in the review study (Figure 1).

In summary, from the articles reviewed (Table 3) that evaluated ventilation efficiency, four did so in obese children [15, 23–25]; six did so after the correction of a Tetralogy of Fallot (TF) [13, 21, 26–29], one did so with Down syndrome children [30], one did so with a bronchial obstruction [31], and only one study evaluated ventilation efficiency in children with sickle cell disease [17]. Other authors aimed to obtain normative data for ventilatory efficiency in healthy children [14, 16, 32, 33]. Table 3 presents the synthesis of the clinical trials included in this study, describing the type of study, objective, and conclusion.

## 4. Discussion

In the physiopathological context, the analyzed studies applied the cardiopulmonary exercise test to the pediatric population aiming to identify abnormalities in the cardiorespiratory system during exercise stress. Exercise stress testing is known as an instrument for the diagnosis of dyspnea and exercise intolerance in children through the analysis of metabolic and cardiorespiratory features during progressive physical effort. Currently, the measure of exercise capacity has been considered part of a multidimensional evaluation, for it evaluates objectively the cardiac, pulmonary, muscular, and metabolic systems, and is considered the gold standard for evaluating exercise intolerance.

The VE/VCO_2_ index is a parameter related to exercise and has been gaining prominence in the management of different clinical conditions. The present review identified the analysis of VE/VCO_2_ in situations regarding healthy children, asthmatic children, children with cystic fibrosis, cardiovascular disease, obese children, adolescents in puberty, premature babies, neurological diseases, and sickle cell disease (SCD) and was also used as a method of evaluation, monitorization, and prognosis, as discussed below. There is not a defined line of research on this subject; the studies show specific, isolated difficulties and problems, and these complicate the comparisons of methodologies and results.

## 5. Ventilatory Efficiency in Children without Diagnosis, Classified as Healthy, in Puberty, and Premature

The first published studies on the subject “VE/VCO_2_ index” were conducted in healthy children and children with cardiopathies. The first study (1987) analyzed the pulmonary ventilation and gas exchange in children who were anesthetized with halothane, enflurane, and isoflurane. The results showed that the ventilatory efficiency was slightly better with enflurane due to the smaller VE/VCO_2_ index value in comparison to the halothane and isoflurane [20]. The next year, with the purpose of establishing normative data through a specific protocol (the James protocol) using a stationary bicycle, 151 North American children (70 girls and 81 boys) were evaluated. It was established that the anaerobic threshold occurred when there was an isolated increase in the VE/VO_2_ without a change in the VE/VCO_2_ [33].

Ten years later, Nagano et al. (1998) studied ventilatory control during exercise in healthy subjects. The results showed that, to keep the PaCO_2_ regulated, children of a lower age, in comparison to older children, would present less tachypnea during exercise to eliminate a certain amount of CO_2_. Therefore, the age of the child should be considered while investigating respiratory control during exercise in pediatric care [32]. Following the same line of questioning, also aiming to analyze the ventilatory response to exercise, 100 prepubescent children executed the submaximal exercise test on treadmills using the modified Balke protocol. For the male subjects, the VE/VCO_2_ index decreased during progressive exercise but remained unaltered for the female subjects. There were differences regarding age and sex in some aspects of the ventilatory response within the pediatric age range [16], which corroborates with the anterior study [32].

In 2010, Prado et al. [37], with 25 healthy children and 20 healthy adults, tested the hypothesis that children presented different responses in the cardiorespiratory and metabolic parameters during the progressive maximal exercise test in comparison with adults. After conducting the progressive test with treadmills up to the point of exhaustion to determine the maximal anaerobic capacity and the ventilatory anaerobic threshold, the authors concluded that the cardiorespiratory and metabolic responses during the progressive exercise test were different in children and adults. Specifically, these differences suggest that the children have less efficient cardiovascular and respiratory systems. However, the children presented a higher metabolic efficiency during the exercise test. The study concludes that, in spite of the differences observed, the children showed similar levels of high exercise capacity when compared to adults [37]. The studies above are relevant in investigating respiratory efficiency, as they recognize the efficient response of this parameter of evaluation and reinforce the concept that age and sex are relevant in evaluating respiratory function: a fact that is also true for children with diseases [17].

Guerrero et al. (2008) evaluated the ventilatory response in 84 children during a cycle-ergometer test to determine if gender influenced ventilatory efficiency. The test started at 25 W (Watts) and increased 10 W each minute. The maximum power output was different between sexes, and the difference was significant for the MV and VCO_2_ and was moderately significant for VE/VCO_2_ [40]. The same results were observed by Lintu et al. (2014) with the objective of obtaining reference values for cardiorespiratory capability, respiratory function, and hemodynamic responses during and after the maximal stress test on a stationary bicycle in children. The authors observed that, in 140 children (69 girls) between the ages of 9 and 11, there was no difference in the VE/VCO_2_ index between boys and girls during the test [9].

There were four clinical trials that studied ventilatory efficiency in childhood obesity [15, 23–25]. The first study, 60 children (30 obese and 30 control) paired by age, sex, and height, aged from 6–17 years, completed the exercise test using a treadmill. The ventilatory efficiency did not differ between the groups during the exercise or the recovery period, although the metabolic expenditure was higher in the obese children [25]. Kaufman et al. (2007) investigated the effect of an aerobic training program of eight weeks on the ventilatory threshold and the ventilatory efficiency of 20 children. The authors found that training with aerobic exercise may help reverse the decrease in cardiopulmonary function, which has been observed over time in these children [15]. Another similar study associated diet with aerobic training in 38 obese children observed that the children had a reduction in body weight. Those who completed only the diet without the associated aerobic training did not improve the VO_2_ maximum and the VE/VCO_2_. The group that took part in both interventions, however, showed an increase in the VO_2_ maximum and ventilatory efficiency. The authors' conclusions were that ventilatory efficiency is reduced in obese children and that diet associated with exercise training improved the VE/VCO_2_ index and cardiorespiratory capability during progressive exercise [23]. However, McMurray and Ondrak (2011) [24] and Marinov et al. (2002) [25] did not identify differences in the VE/VCO_2_ values while studying a specific exercise program in 73 obese children paired with 73 healthy children, which establishes a scientific controversy relating to the subject.

## 6. Ventilatory Efficiency and Children with Respiratory Diseases (Asthma, Bronchial Obstruction, and Cystic Fibrosis)

Physical exercise training programs have been increasingly recommended for children with respiratory diseases, especially asthma and cystic fibrosis. However, it is not clear what the ideal respiratory pattern to be aimed for is during physical activities in each disease. Ceugniet et al. (1996), while studying 15 asthmatic aged 12–19 yrs divided into a control group and a low-frequency respiration group, aimed to lower their respiratory frequency by 40% during exercise. The authors concluded that the respiratory pattern might be altered during exercise without an increase in dyspnea. Furthermore, as you lessen the respiratory frequency during the exercise, you also diminish the VE/VCO_2_ by 19%. The author calls our attention to the fact that, while you direct the respiratory patterns during rehabilitation exercises, you must evaluate the patient individually to determine the desired pattern and avoid hypercapnia and hypoxia [41]. Even though the importance of this subject is well known, there are not many studies in the field, which makes a comparative analysis difficult.

In the cases related to exercise-induced bronchial obstruction, 11 children were evaluated with the Bruce protocol on a treadmill, with the objective of verifying the characteristics of their ventilatory response to exercise. The children presented lesser oxygen (O_2_) consumption when compared to healthy individuals. Three patients with bronchial obstructions developed relative hypoventilation during incremental exercise, identified by the increase in the end-tidal carbon dioxide pressure (PETCO_2_) and a decrease in the VE/VCO_2_ observed at the final stages of the test. These results demonstrate that some bronchial obstructions may evolve into bronchoconstriction during exercise [31] and, therefore, their test must be conducted safely with professional monitoring.

In cystic fibrosis, while studying the chemosensitivity of CO_2_ and ventilatory efficiency during exercise in 39 children with the disease and a healthy control group, the hypercapnic ventilatory response showed a decline related to age in both groups. In the healthy group, there was an inverse relation between age and the VE/VCO_2_ index. In other words, as age increased, there was a decline in the VE/VCO_2_ index [17]. The findings show that, even in healthy children, there is a proportional decline in ventilatory efficiency with age. While developing a multivariate model for predicting mortality in 127 adolescents with cystic fibrosis, Hulzebos et al. (2014) considered the VE/VCO_2_ index a strong mortality predictor in the studied population. In clinical practice, this may be a useful tool for detecting patients with high mortality risks and providing them with additional preventive therapy [8].

## 7. Ventilatory Efficiency and Children with Other Diseases (Cardiopathies, Sickle Cell Disease, Congenital Central Hypoventilation Syndrome, and Pulmonary Hypertension)

Some authors studied ventilatory efficiency in other clinical situations and in different respiratory illnesses [10, 11, 15, 16, 18, 22, 28, 32, 36, 42].

The efficiency of spontaneous ventilation and the ventilatory efficiency obtained by the VE/VCO_2_ index were investigated in 18 babies and children with congenital cardiac disease, while they were anesthetized with halothane. The subjects were divided into two groups: (1) pulmonary hyperperfusion and shunt from left to right and (2) pulmonary hypoperfusion and shunt from right to left. MV and tidal volume (TV) were higher in group 2, while dynamic pulmonary compliance, pulmonary resistance, and alveolar ventilation were, in the same magnitude, higher in both groups. The VE/VCO_2_ and VD/VT index were higher in children with lower pulmonary blood flow than in children with higher blood flow, indicating a less efficient gas exchange in children with shunt from right to left [22]. Also in cardiology, the ventilatory response to exercise after intracardiac correction of TF was analyzed in a group of 13 children ranging in age from 7–13. It was observed that the VCO_2_, VE, VE/VO_2_, and VE/VCO_2_ were high during the progressive exercise in comparison to the healthy control group of the same age, height, and sex. Therefore, cardiopathic children who are clinically stable may present abnormalities in pulmonary gas exchange, which is compatible with a diminished distribution of pulmonary perfusion expected after the correction of TF procedure [28].

A multicentric prospective study also analyzed a group of 272 (158 boys with a mean age of 14.3 ± 3.3) patients after the correction of TF. The patients underwent cardiac magnetic resonance imaging for the evaluation of ventricular functioning, and a metabolic stress test was later performed. The female patients presented a decreased ejection fraction of the right ventricle, an inferior muscle mass of the right ventricle, lower oxygen consumption, higher VE/VCO_2_ index, and a reduction in the heart rate peak value. The parameters of the left ventricle did not differ between sexes [13].

In the same year, Lee et al. 2013, aiming to obtain normality values for Korean children and adolescents through cardiopulmonary testing, studied 76 healthy children and adolescents who were submitted to the modified Bruce protocol. The maximal oxygen consumption (VO_2_max) and the metabolic equivalent (METmax) were higher in boys than in girls. The VE/VCO_2_ index did not differ between boys and girls. The data of the cardiopulmonary test did not differ between boys and girls in the younger age group (ages 10–14). However, in the older age group [15–19], the boys presented higher VO_2_peak and METmax values and lower VE/VCO_2_ values than the girls [14].

Between 2002 and 2011, 50 cardiopathic children (24 girls (18 with palliated single ventricle) with a mean age of 15) were submitted to the exercise test. The VO_2_peak < 50% from the normal value was associated with children with biventricular circulation but not with those with a palliated single ventricle. In the same way, VE/VCO_2_ ≥ 34% was associated with children with biventricular circulation but not with children with a palliated single ventricle [35]. Recently, 101 (mean age = 12.1) patients with single ventricle disease were submitted to a cardiopulmonary exercise test. The results showed that the VE/VCO_2_ index was 127% ± 30% of the expected value [11].

A different clinical situation investigated was the ventilatory response of children with congenital central hypoventilation syndrome (CCHS). Researchers used motorized cycle ergometers in frequencies from 6 to 60 rpm. With passive leg movement, it was observed that there was a bigger increase in pedaling frequency in the patients with CCHS than in the healthy controls, while the TV increased in both groups. At 60 rpm, there was an increase of VO_2_ in both groups; VE/VO_2_ and VE/VCO_2_ were increased in the patients with CCHS and were constant in the control group. The passive movement of the leg brought on a normalization of the PETCO_2_ in individuals with CCHS [18].

However, since this study stands alone in its field, new investigations must be made for a complete understanding of the ventilatory behavior in children with CCHS.

SCD is frequently studied with the objective of improving the management of the disease, especially in pediatrics. It is known that adults affected by this disease develop restrictive pulmonary insufficiency, an increase in dead alveolar space, and hypoxemia. These factors, together with the increased anaerobic metabolism, are responsible for hyperventilation during physical exercise. The authors evaluated pulmonary function in an ergometric test in children with SCD. They compared the results to the control group and found that the children with the illness had a higher respiratory response to exercise. This event is, in part, caused by the increase in physiological dead space and the low hemoglobin count. The increased dead space is a result of the sickled cells, which causes a low capillary perfusion of the ventilated alveoli [39].

While comparing the six-minute walking distance (6MWD) with the performance during the cardiopulmonary exercise test in 41 children with pulmonary hypertension, it was demonstrated that the maximal oxygen consumption and distance in the 6MWD were decreased to 31.5 ± 12.2% and 47.7 ± 16.7% of the predicted values, respectively (*P* < 0.0001 for both). The maximal oxygen consumption and the oxygen consumption at the anaerobic threshold were correlated with the distance covered in the 6MWD, while the ventilatory efficiency parameters at the anaerobic threshold (such as VE/VCO_2_) and distance covered in the 6MWD were found to be inversely correlated [42].

## 8. Other Considerations

The present study reviewed the methodological quality of the studies considering the VE/VCO_2_ index, as well as the different outcomes found in the studies. Some limitations identified in the studies are of great methodological importance, for example, a lack of a control group for comparing results. Some studies evaluated a small number of patients, which restricts the statistical analysis, considering the numerous parameters studied. A heterogenic group of patients was also a methodological difficulty discovered, since this interferes in the physiopathological analysis of the individuals submitted to exercise. The most significant limitation found when analyzing the studies was a lack of a specific line of research on this theme. If a line of research was to be established, it could strengthen the findings, giving more credibility to the results that are being published.

Regardless of the statements above, the cardiorespiratory and metabolic responses during the progressive exercise test in children, when compared to adults, demonstrated that children possess lower cardiovascular and respiratory efficiency (but higher metabolic efficiency) during the test; however, they present similar levels of exercise capacity [38].

## 9. Conclusion

The ventilatory efficiency in pediatrics is a study area with limited publication and knowledge. There still is no well-defined line of research for investigating the ventilatory efficiency in pediatrics. The studies found were conducted in different clinical situations: they present controversy and methodological divergences. However, it seems consensual that the VE/VCO_2_ index, during increasing physical exercise, decreases progressively and rises again only at the end of the stress. Furthermore, this relation may be used to characterize ventilatory response and exercise capacity. However, few studies evaluate the cardiorespiratory system through ventilatory efficiency in children. It has been argued that a high VE/VCO_2_ index may be associated with a bad prognosis, for it is related to a diminished capacity of pulmonary perfusion and cardiac output. Furthermore, factors, such as age and sex, appear to interfere with the behavior of the VE/VCO_2_ index.

## Figures and Tables

**Table 1:** Search strategy on PEDro, MedLine, and PubMed databases.

PEDro	Advanced search: title: VE/VCO_2_
	Specialty: pediatrics

PubMed	VE/VCO_2_ adolescents children^∗^
	VE/VCO_2_ adolescents^∗^
	VE/VCO_2_ children^∗^

MedLine	VE/VCO_2 _adolescent children^∗^

^∗^In all search strategies, the terms were associated (“AND”). VE/VCO_2_: minute ventilation (MV)/production of carbon dioxide (VCO_2_).

**Table 2:** Title and methodological quality of the trials on the VE/VCO_2 _index applied to pediatrics, according to the PEDro database.

Title	Score
Aerobic-exercise training improves ventilatory efficiency in overweight children	5/10
Weight loss associated with exercise training restores ventilatory efficiency in obese children	4/10
Effects of aerobic training in adolescents with Down syndrome	3/10

Source: http://www.pedro.org.au/. VE/VCO_2_: minute ventilation (MV)/production of carbon dioxide (VCO_2_).

**Table 3:** Summary of the selected studies from PEDro, MedLine, and PubMed.

Study	Type of study	Objective	Sample	Conclusion
[9]	Clinical trial and prospective	To provide reference values for cardiorespiratory ability, respiratory function, and hemodynamic responses during and after the maximal exercise test on stationary bicycles in children.	140 children (69 girls), ages 9–11.	The boys' indicators of cardiorespiratory capability surpassed the girls'. There was no difference in the VE/VCO_2_ index between the sexes.

[8]	Clinical trial and prospective	To use parameters to develop a multivariate model for predicting mortality in adolescents with CF.	127 adolescents with CF (mean age = 12.7 ± 0.9).	The joint evaluation of BMI, FEV_1_%, and VE/VO_2_ is a strong predictor of CF adolescent mortality.

[34]	Clinical trial	To create prediction equations for the CPT variables based solely on patients with Fontan circulation.	411 patients were submitted to the CPT; 166 in the maximal exercise stress test.	The VE/VCO_2_ and VE/VO_2_ indices at the anaerobic threshold had an inferior outcome in the validation cohort. Six from the eight prediction equations for the CPT variables proved valuable and were validated.

[11]	Clinical trial and prospective	To evaluate the exercise capacity in cardiac patients and compare the results between the two corrective surgical techniques.	101 patients submitted to the CPT.	VE/VCO_2_ was 127% ± 30% from the predicted value. Results showed a reduced exercise capacity in patients with Fontan correction.

[12]	Clinical trial and controlled	To evaluate the correlation between ventilatory efficiency and the functional capacity in pediatric patients with PH.	76 children and young adults with PH were submitted to 258 cardiopulmonary exercise tests.	The VE/VCO_2_ index was higher in PH patients, in comparison to the control group. The index was the highest in 12 patients with the worst prognosis (nine deceased, three pulmonary transplants).

[13]	Clinical trial, multicentric and prospective	To evaluate the ventricular function and metabolic effort test between sexes.	272 patients (158 boys (mean age = 14.3 ± 3.3)) with TF.	Women had a more inferior performance than men regarding the systolic function of the right ventricle, evaluated with cardiac magnetic resonance and exercise capacity, in addition to presenting a higher VE/VCO_2_ index.

[14]	Clinical trial and retrospective	To supply reference data for the CPT variables in children and adolescents.	76 healthy children and adolescents who underwent the PCT test using the modified Bruce protocol.	The VE/VCO_2_ index did not differ between boys and girls. In the group aged from 15–19 years, the boys presented higher values of VO_2_peak and lower values of the VE/VCO_2_ index than the girls.

[35]	Clinical trial and prospective	To evaluate the association of the VO_2_peak <50% of the predicted value during the CPT in cardiac children with death risk or deterioration of cardiac function.	50 children (24 girls (mean age = 15, range = 13–17)), 18 with a single ventricle in palliative care.	VE/VCO_2_ ≥ 34 was associated with children with biventricular circulation but not with children with a single ventricle in palliative care.

[24]	Controlled	To evaluate the ventilatory dynamics in obese and nonobese children.	73 overweight children were compared using parameters of age, sex, and height.	The VE/VCO_2_ index was similar between groups.

[36]	Clinical trial and cross-sectional	To investigate the efficiency of oxygen absorption in children with CHD.	31 adolescents with CHD (16 with repaired Fontan and 15 with TF) underwent the CPT.	Ventilatory efficiency may be a valid parameter, regardless of the cardiorespiratory capability of children with coronary disease.

[37]	Clinical trial and cross-sectional	To verify if children present different responses to the evaluated cardiorespiratory and metabolic parameters during the maximal progressive exercise test when compared to adults.	25 healthy children (15 males, 10 females) (mean age 10.2 ± 0.2) and 20 healthy adults (11 males, 9 females) (mean age = 27.5 ± 0.4).	During the CPT, at the ventilatory anaerobic threshold, the HR, VO_2_, RF, VCO_2_, VD/VT, VE/VO_2_, VE/VCO_2_, and PETO_2_ responses were higher in children when compared to adults.

[38]	Clinical trial, controlled and randomized	To verify in obese children if (1) ventilatory efficiency is diminished during progressive exercise, (2) loss of weight through diet improves ventilatory efficiency, and (3) diet associated with exercise training improves ventilatory efficiency.	38 obese children. Ten healthy children were included as a control group. All children underwent CPT.	Ventilatory efficiency was lower in obese children who presented weight loss through progressive exercise.

[29]	Cross-sectional	To determine if, in patients with corrected TF, there was improvement in the poor pulmonary blood flow distribution, after a surgical procedure, during exercise.	17 patients with corrected TF and residual stenosis of the pulmonary artery who were forwarded to a balloon angioplasty.	The patients with the balloon angioplasty presented a better VO_2_peak and more efficient gas exchange during exercise.

[15]	Clinical trial and randomized	To investigate the effect of an eight-week exercise training course on the ventilatory threshold and ventilatory efficiency in overweight children.	20 overweight children underwent the progressive exercise test. They were split randomly into eight weeks of cyclism or a control group.	Aerobic exercise training may help to reverse the loss of cardiopulmonary function observed throughout time in overweight children.

[25]	Controlled	To verify ventilatory efficiency and the effort perception in obese and nonobese children submitted to a standard exercise load.	60 children (aged 6–17) were divided into two groups: 30 obese and 30 healthy individuals.	The VE/VCO_2_ index did not differ between groups. In the studied population, the metabolic cost during exercise was higher in the obese group when compared to the control individuals.

[16]	Cross-sectional	To verify the differences in the ventilatory response to exercise in children and preadolescent individuals.	100 children divided into two groups: 10 years old and 13 years old.	There are differences in age and sex, in some aspects, of the ventilatory responses in pediatric subjects.

[19]	Controlled	To evaluate intra- and interrater reliability and the validity of the ventilatory threshold parameter in children.	35 premature children aged 6–12 years, and 20 term born controls.	The TV was considered the valid parameter for establishing aerobic capacity.

[32]	Cross-sectional	To investigate the relation between age and respiratory control in pediatrics.	80 children aged 6.4–17.6 years (42 males and 38 females).	Younger children, while eliminating CO_2_ and regulating PaCO_2_, presented less tachypnea during exercise when compared to older children.

[31]	Controlled	To document the ventilatory response to exercise in patients with exercise-induced bronchial obstruction.	11 children with bronchial obstructions.	Patients with a bronchial obstruction develop bronchoconstriction during exercise.

[17]	Cross-sectional and controlled	To determine the relation between the PCO_2_ and CO_2_ receptors and the respiratory response during exercise in healthy children and children with CF.	16 healthy children and 16 children with CF in phase 1 and 28 healthy children and 23 children with CF in phase 2.	The younger children ventilate more during exercise than the older children because they regulate the PaCO_2_ at a lower level. The hypercapnic ventilatory response may be reduced in the presence of airway obstructions, being that a low hypercapnic ventilatory response may permit an exercise-induced hypercapnia in some patients with CF or advanced pulmonary disease.

[18]	Controlled	To evaluate the MV during exercise in a cycle ergometer in children with central hypoventilation syndrome compared to a control group.	6 children with CCHS and 6 healthy children.	The passive movement of the legs in pedaling increased the MV in both groups. The passive movement of the legs normalized the PetCO_2_ in patients with hypoventilation syndrome.

[30]	Clinical trial and controlled	To evaluate the effects of aerobic training on adolescents and young adults with Down syndrome.	14 individuals with Down syndrome (mean age = 17.7).	Even though the training program did not improve aerobic capacity, it improved walking capacity.

[28]	Cross-sectional and controlled	To test, through exercise, the poor perfusion after intracardiac repair of TF.	13 children aged from 8–18 years, clinically stable (Class I) with 7–14 years post-op of the intracardiac repair of the TF, and 16 children in the control group.	The clinically stable children may present abnormalities in gas exchange, which is compatible with the slightly poor pulmonary perfusion expected 7–14 years after the surgical repair of TF.

[39]	Controlled	To evaluate the increase in aerobic metabolism in SCD patients.	34 patients with SCD, and 16 control individuals.	Children with SCD have an increased ventilatory response during exercise caused, partially, by the physiological increase of dead space and low hemoglobin. The increase in pulmonary dead space may be the result of sickled cells, which affect the capillary perfusion of the ventilated alveoli.

[33]	Cross-sectional	To establish normative data for untrained children through the James protocol in stationary bicycles.	151 North American children aged from 7–12 years.	The data may be used in the evaluations of preadolescents in North America.

[20]	Cross-sectional	To evaluate the pulmonary ventilation and gas exchange in anesthetized children with halothane, enflurane, and isoflurane.	24 children who were submitted to surgical procedures.	Although the MV was lower with the enflurane, the ventilatory efficiency was similar between the anesthetics.

[22]	Cross-sectional	To investigate the ventilatory efficiency during the use of halothane.	18 babies and children with congenital heart disease divided into two groups (1) hyperperfusion and shunt from left to right and (2) hypoperfusion and shunt from right to left.	The VE/VCO_2_ and VD/VT were higher in children with diminished pulmonary blood flow, indicating a less efficient gas exchange in children with shunt from right to left.

CO_2_: carbon dioxide; CCD: congenital cardiac disease; RF: respiratory frequency; PaCO_2_: carbon dioxide partial pressure; PetCO_2_: end-tidal carbon dioxide pressure; MV: minute ventilation; VCO_2_: partial pressure of CO_2_ in arterial blood; VD/VT: dead space/tidal volume ratio; VE/VCO_2_: ventilatory efficiency index; VE/VO_2_: ventilatory equivalent for oxygen; TV: tidal volume; HR: heart rate; BMI: body mass index; CF: cystic fibrosis; PH: pulmonary hypertension; CPT: cardiopulmonary test.

## Abbreviations

CO_2_: Carbon dioxide
HR: Heart rate
RF: Respiratory frequency
PetCO_2_: End-tidal carbon dioxide pressure
PetO_2_: End-tidal oxygen pressure
PaCO_2_: Carbon dioxide partial pressure
VCO_2_: Partial pressure of CO_2_ in arterial blood
VO_2_: Partial pressure of O_2_ in arterial blood
VE/VCO_2_: Ventilatory efficiency index
VE/VO_2_: Ventilatory equivalent for oxygen
VO_2_peak: Peak oxygen consumption
VO_2_max: Maximal oxygen consumption
MV: Minute ventilation
TV: Tidal volume
PEDro: Physiotherapy Evidence Database
MEDILNE: Medical Literature Analysis and Retrieval System Online
PubMed: US National Library of Medicine National Institutes of Health
CCHS: Congenital central hypoventilation syndrome
METmax: Metabolic equivalent
SCD: Sickle cell disease
TF: Tetralogy of Fallot
6MWD: Six-minute walking distance
BMI: Body mass index
CPT: Cardiopulmonary test
PH: Pulmonary hypertension
FEV_1_%: Forced expiratory volume in 1 second
CCD: Congenital cardiac disease
VD/VT: Dead space/tidal volume ratio
Rpm: Revolutions per minute.
